# The impact of mineralocorticoid receptor antagonists on fat mass and fat metabolism

**DOI:** 10.1007/s40618-026-02909-0

**Published:** 2026-05-22

**Authors:** Baris Afsar, Rengin Elsurer Afsar, Geetha Maddukuri

**Affiliations:** 1 School of Medicine, Department of Nephrology, Suleyman Demirel University, SuleymanDemirel University Hospital, Cunur, 32260 Isparta, Turkey; 2 School of Medicine, Department of Nephrology, Saint Louis University, SSM Health Saint Louis University Hospital, St Louis, MO USA; 3 Division of Nephrology and Hypertension, VA Saint Louis Health Care System, St Louis, MO USA

**Keywords:** Eplerenone, Finerenone, Fat, Mineralocorticoid receptor antagonists, Obesity, Spironolactone

## Abstract

Introduction: Obesity is a chronic, multifactorial condition which is a global health challenge. In general, obesity is defined by excessive fat accumulation and associated with the risk of numerous conditions, including type 2 diabetes mellitus (T2DM), cardiovascular disease, heart failure, and metabolic dysfunction-associated steatotic liver disease, and some types of cancer contributing to increased morbidity and mortality. Mineralocorticoid receptor antagonists (MRAs) are used for a long time for management of cardiovascular disease and non-steroidal MRAs also showed kidney protection. Apart from these, there is growing literature showing that MRAs effecting fat mass and fat metabolism. In this review, we summarised the effects of MRAs on fat mass and fat metabolism.

Results:In general, studies showed that MRAs reduced fat mass in various organs which is associated decreased oxidative stress, inflammation, and insulin resistance. Importantly, MRAs increased adipose tissue browning and decrease white adipose tissue mass. Up to now, the studies showed no safety signals and these favorable effects are independent of blood pressure reduction. Discussion A part from hemodynamic effects, MRAs have beneficial impacts on fat mass and fat metabolism. Currently, the underlying mechanisms for these effects are not clear

Conclusions: Preliminary studies have shown that MRAs have favorable impact on fat metabolism and fat mass. Studies needed to highlight underlying mechanisms..

## Introduction

Obesity which is a global health challenge is a chronic, multifactorial condition. In general, obesity is defined by excessive fat accumulation and associated with increased risk of numerous conditions, including type 2 diabetes mellitus (T2DM), cardiovascular disease, heart failure, and metabolic dysfunction-associated steatotic liver disease [1]. Healthy lifestyle modification, pharmacological and/or surgical interventions are main measures for prevention and treatment of obesity but the efficiency of these measures are not satisfactory [2, 3]. This necessities more comprehensive approach and novel treatment options.

Contrary to previous thoughts, adipose tissue is not a passive lipid storage depot but it is an active endocrine organ. Adipose tissue secretes adipokines (e.g., leptin, adiponectin, resistin), inflammatory cytokines (e.g., tumor necrosis factor-alpha [TNF-α], interleukin-6 [IL-6]) which exert autocrine, paracrine, and endocrine effects. In addition, during obesity, adipose tissue expansion is maladaptive and contributes to increased oxidative stress [4, 5]. Furthermore, adipose tissue is not homogeneous but composed of white, brown and beige (brown in white) which have distinct functions [6].

Aldosterone classically exerts its effects via mineralocorticoid receptors (MR) which are present in renal epithelial cells. Recently, there is increased discussion regarding the relationship between increased mineralocorticoid activity and obesity. Studies have shown that aldosterone levels and body mass index (BMI) are correlated [7, 8]. Interestingly, epicardial fat tissue which is considered to represent the true visceral fat depot, was increased in patients with primary hyperaldosteronism (PA) compared to patients with essential hypertension and epicardial fat tissue was associated with plasma aldosterone concentrations [9]. In addition, positive association was demonstrated between increasing BMI and clinically confirmed primary aldosteronism (PA) [10]. Additional studies have shown a significant association between BMI and plasma and/or 24-h urinary aldosterone concentration (without clinical diagnosis of PA) in normo to hypertensive patients [8, 11]. In the Framingham Offspring Study of 1473 subjects, increasing tertiles of aldosterone were independent predictors of the development of metabolic syndrome over the next 3 years [12]. On the other hand, weight loss in 285 obese young adults showed significant reductions in aldosterone concentration in those with metabolic syndrome at baseline [13]. Furthermore, adipocytes can synthesize and secrete aldosterone [14, 15]. Additionally, high fat diet induces Renin-Angiotensin Aldosterone (RAS) system activation [16]. ItIt was also demonstrated that administration of mineralocorticoid receptor antagonists (MRAs) in murine models of obesity reduced white adipocyte dysfunctions, improved glucose tolerance, and induced browning of white adipose tissue [5, 17].

All these evidence suggest that mineralocorticoid receptor blockage may be important and feasible intervention in reducing fat mass, obesity and related metabolic disturbances. To this end, in this narrative review; we summarized the studies specifically investigating the impact of mineralocorticoid receptor blockage on fat tissue accumulation and consequent metabolic effects. We also identify knowledge gaps and future study proposals.

## Relationship between mineralocorticoid receptor antagonists on fat mass and fat metabolism

We summarized both preclinical and clinical studies specifically investigating the relationship between use of MRAs with fat mass in Tables 1 and 2 respectively. Experimental studies showed that treatment with MRAs decreased fat content in endothelium [18] in inguinal and intersacpular area [19], in liver [20–22], in aortic arch [23], in muscle [24], and in myocardium [25]. These medications also cause browning of adipose tissue [19, 20, 26] and decrease white adipose tissue [17]. They also reduce inflammation and oxidative stress in adipose tissue [5].

**Table 1:** Summary of experimental studies specifically investigating the impact of mineralocorticoid receptor antagonists on fat mass and related mechanisms

References	Methods	Effect on fat mass	Related mechanisms
Wei et al. [18]	-Ren2 rats (overexpressing renin with increased ANG II and aldosterone levels) assigned to vehicle-treated or Spironolactone treated groups (0.24 mg/d) or for 21 day	- Increased lipid accumulation in media and endothelial cells (Oil red O staining) in Ren2 aortas attenuated with Spironolactone	-Spironolactone reduced vascular oxidative stress (decreased 4-Hydroxynonenal,3-nitrotyrosine, NADPH oxidase, NADPH oxidase 2, Ras-related C3 botulinum toxin substrate 1) -Spironolactone increased expression of anti-apoptotic molecule Bcl-xL in aorta -Spironolactone partially improved mitochondrial abnormalities (swelling, cristae disruption, and reduced matrix density and decreased cytochrome c release to cytoplasm -No change in BPs observed
Armani et al. [19]	C57BL/6 mice divided into 4 groups: (i) with normal diet (10% kcal as fat) (ii) with high-fat diet (HFD) diet (45% kcal as fat (iii) HF + Drospirenone (DRSP; 6 mg/kg/d) (ıv) HF + Spironolactone (20 mg/kg/d) for 90 days -Inguinal subcutaneous fat depots assessed by magnetic resonance spectroscopy -Abdominopelvic fat was assessed by MRI analysis -Murine 3T3-L1 pre-adipocytes d treated with Aldosterone (10^–8^ M), DRSP (10^–5^ M), or Spironolactone (10^–5^ M) for 7 days	-HFD increased body weight compared with normal diet -HFD increased weight of the perivescical, parametrial, inguinal, and interscapular fat pads which all were decreased by Spironolactone -Expression of brown adipocyte marker genes cell death inducing DNA fragmentation factor, a subunit-like effector A (CIDEA), beta-3 adrenergic receptor (Adrb3), and UCP-1 in inguinal fat increased with DRSP and Spironolactone -Both MRAs decreased the size of cytoplasmic lipid droplets, increased water content of inguinal fat compared with the HFD suggesting browning of adipose tissue -In 3T3-L1 pre-adipocytes, MRAs significantly increased PGC1-α, CIDEA, Adrb3, and UCP-1 transcript levels	- Spironolactone or DRSP prevented the HFD induced increase in blood glucose after intraperitoneal glucose tolerance test -DRSP and Spironolactone reduced transcript levels of Leptin and adenylate cyclase 5 which are enriched in white adipocytes
Zhu et al. [32]	C57BL/6 J mice treated with (i)Normal diet (10% kcal as fat) (ii) HFD 60% kcal as fat) (iii): HFD + daily subcutaneous injections of spironolactone (20 mg/kg/d for 3 weeks -Mouse podocyte cell are used for in vitro experiments -Visceral fat mass was measured and obesity index were calculated	-Abdominal visceral fat index increased by HFD but significantly reduced by spironolactone	- Serum triglyceride and cholesterol increased by HFD significantly decreased by Spironolactone
Habibi et al. [20]	-C57BL6J mice few with chow or a western diet (WD) composed of saturated fat (46%) sucrose (17.5%), and fructose corn syrup (17.5%), with or without Spironolactone (1 mg/kg/day) for 16 wk -After 16 wk. body composition analysis performed by quantitative MRI -Liver and epi-abdominal fat samples for histology	-WD increased body weight, liver weight, total fat mass, liver fat and epi-abdominal fat without alterations in lean mass -Spironolactone prevented increase of hepatic lipid content -In epi-abdominal fat tissue, spironolactone increased browning of white adipose tissue along with increased brown fat genes encoding for UCP-1, Adrb3	-WD increased expression of hepatic fatty acid transporter protein CD36, Fabp1 which were prevented by Spironolactone -WD increased hepatic expression of IL-6, TNF-α CD68 and CD86, MMP-2 and MMP-9 Which were significantly blunted by Spironolactone
Hulse et al. [24]	- C57BL6J mice few with chow or a western diet (WD) composed of saturated fat (46%) sucrose (17.5%), and fructose corn syrup (17.5%), with or without spironolactone (1 mg/kg/day) for 16 wk -MRI used for body composition analysis -Muscle bıopsy done from soleus muscle	- WD consumption increased both serum and soleus FFA levels, as well as soleus intramyocellular lipid content, were prevented by Spironolactone	-In soleus muscle, WD diet increased expressions of CD36, Fabp3 and fatty acid which were prevented by Spironolactone -In soleus, mitochondrial transcription factor cytochrome c oxidase I and cytochrome b (markers of mitochondrial biogenesis and Metabolism) were reduced by WD which were blunted by Spironolactone
Marzolla et al. [26]	- C57BL/6 J mice divided into 3 HFD, HFD + Spironolactone 0,165 gr /kg and HFD + Finerenone 0.1 g of Fine/kg of diet for 12 weeks - WAT and activate interscapular brown adipose tissue (iBAT) used for histological analysis	-Spironolactone did not affect lipid droplet size in interscapular BAT compared to HFD -Finerenone increased interscapular BAT fed with a very HFD diet - In iBAT, thermogenesis-related gene expressions of UCP-1, PGC1-α), Adrb3 increased by Finerenone vs. HFD group -Spironolactone did not alter the expression of these genes	- Finerenone reduced insulin resistance in mice fed with HFD -Expression of white adipocyte-specific marker genes (leptin and adyc5) in iBAT of HFD + Finerenone group was lower compared with HFD (with no differences between HFD + Spironolactone
Hirata et al. [5]	- C57BL/6 J Obese ob/ob and db/db mice were treated with control diet with and without Eplerenone (1 mg/g) for 3 weeks 3T3-L1 adipocytes were treated with aldosterone with and without Eplerenone - Epididymal fat pad used for histological analysis	- In ob/ob and db/db mice, marked reduction of hypertrophic adipocytes observed in Eplerenone group vs. Control group	- F4/80 staining showed increased macrophage infiltration increased in adipose tissue of obese mice which is reduced by Eplerenone - Eplerenone decreased adipose tissue mRNA level of CD11c (M1 macrophage marker) -In adipose tissue Eplerenone decreased the expressions of TNF-α, MCP-1, and IL-6 - In db/db mice, Eplerenone decreased Leptin expression, and reversed the reduction in mRNA expressions of PPARγ and adiponectin - TBARS as a measure of oxidative stress increased, in adipose tissue of ob/ob and db/db mice but significantly reduced by Eplerenone - Aldosterone increased intracellular TBARS levels in cultured 3T3-L1 adipocytes but reversed by Eplerenone - In cultured 3T3-L1 adipocytes, mRNA levels of and NADPH oxidase subunits p22 and p47 increased whereas catalase decreased significantly by Aldosterone whereas such increases were reduced by Eplerenone
Ramírez et al.[25]	-3 groups of rats used (I) Zucker Lean (ZL) rats (control) (II)Non-hypertensive Zucker Diabetic Fatty (ZDF) rats (vehicle) (III) ZDF rats with Eplerenone (25 mg/kg) treated for 16 weeks -After 16 weeks, cardiac structure and function examined -H9c2(2–1) myoblast cell line derived from embryonic BD1X rat heart tissue used for in vitro experiments	-In ZDF rat myocardium numerous cytosolic lipid droplets observed suggesting myocardial steatosis -CD36 increased in the ZDF myocardium -In ZDF myocardium two rate-limiting enzymes of lipid re-esterification, GPAT1 and DGAT1 were up-regulated along with increased TAG content -ZDF hearts accumulated sphingolipids precursors of ceramides such as ketosphingosine and sphingosine -Eplerenone decreased the expressions of CD36 FABP3 myocardial steatosis, GPAT1/DGAT1, and ceramides intermediates -In cultured myocytes, eplerenone mitigated cytosolic lipid accumulation, CD36 and FABP3 expression after HFD	-Eplerenone reduced plasma lipids without significant changes in SBP -In ZDF rats, Eplerenone decreased interventricular septum hypertrophy and ameliorate diastolic dysfunction - Eplerenone reduced cardiac apoptosis both in vivo and in vitro
Wada et al. [21]	Wild type (WT) mice divided into 4 groups: WT1: Regular diet (4.8% fat), WT2: high-fat and fructose diet (HFFD (60% fat, 30% fructose), WT3: Regular diet + Eplerenone (1.67 mg/g), WT4: HFFD + Eplerenone (1.67 mg/g -Transgenic mice (TG) overexpressing the active form SREBP-1c divided into TG1: Regular diet 4.8%(4.8% fat), TG2: HFFD (60% fat, 30% fructose), TG3: Regular diet + Eplerenone (1.67 mg/g), TG4: HFFD + Eplerenone (1.67 mg/g) -All interventions for 12 weeks -Hepatic triglyceride content was determined by triglyceride colorimetric kit -Liver and epididymal fat tissues were obtained for histologic analysis	- Hepatic triglyceride content was significantly increased by HFFD (more in TG mice vs WT mice) and significantly ameliorated by Eplerenone in both genotypes - In epididymal fat tissue, size of adipocytes were higher with HFFD compared to regular diet an significantly decreased by Eplerenone in both genotypes	-Body weight, liver weight, and epididymal fat weight were similar between WT and TG mice under regular diet feeding -HFFD increased fasting glucose, fasting insulin, and the estimated HOMA-R index in both WT and TG mice but these elevations were attenuated with Eplerenone in both genotypes -TG mice fed with HFFD exhibited steatosis with infiltration of proinflammatory cells (polymorphonuclear leukocytes, eosinophils, and macrophages) and these alterations are ameliorated with Eplerenone
Pizarro et al. [22]	-C57bl6 mice induced NASH fed a choline-deficient-amino-acid–defined (CDAA) diet for 22 weeks with and without at a dose Eplerenone of 1 mg/g of diet -Hepatic triglyceride content and hepatic mRNA levels of pro-inflammatory pro-fibrotic, oxidative stress-associated genes assessed	-Eplerenone prevented the increase in liver hepatic triglyceride content and steatosis	-Liver fibrosis assessed by Sirius red staining were reduced markedly with Eplerenone -Eplerenone attenuated the CDAA diet-induced upregulation of fibrosis markers Col1A1, Col3A1 and MMP-2 -No effect of Eplerenone on liver inflammation observed and oxidative stress
Wada et al. [17]	-C57BL/6 mice and db/db mice used for experiments -C57BL/6 mice divided into 4 groups: chow diet, chow diet + Eplerenone 1.67 g/kg diet, HFD (60 kcal% fat diet) and HFD + Eplerenone1.67 g/kg diet, HFD for 12 weeks -Hepatic triglyceride content was determined by triglyceride colorimetric kiT -Body fat composition analyzed by MRI after 12 weeks -Isolated adipose tissues and livers for histologic analysis performed -In obese db/db mice, and their control misty mice Eplerenone administered (1.67 g/kg diet)	Eplerenone did not affect body weights and body fat volumes with chow-diet feeding -HFD induced visceral and subcutaneous fat by feeding was significantly attenuated by eplerenone -Histology of epididymal WAT and subcutaneous WAT showed increased adipocyte size by HFD vs. chow diet, but these changes were suppressed by Eplerenone -Eplerenone did not affect lean mass - Lipid accumulation in the hepatic lobules in HFD mice was significantly reduced in HFD + Eplerenone mice	- Eplerenone did not reduce increased fecal triglyceride levels induced by HFD -Core body temperature was significantly lowered in HFD mice, but maintained in HFD + Eplerenone mice - The oxygen consumption and carbon dioxide productions were remarkably decreased in HFD but restored in HFD + Eplerenone mice -In eWAT, M1 macrophages increased and M2 Macrophages decreased under HFD-fed conditions and these alterations significantly attenuated by Eplerenone -In epididymal WAT and liver of HFD mice, expressions of IL-β, IL-6 and TNF-α and phosphorylation of NF-κB and caspase levels increased vs chow diet but maintained at normal levels in the HFD + Eplerenone mice
Vecchiola [16]	- C57BL/6 J mice with 3 groups Normal diet, HFD, HFD + Eplerenone (60 mg/pellet) 90-day release which delivers 0.67 mg/day of the drug -MRI used for body composition analysis -Epididymal WAT and livers used for histological analysis -SW-872 (ATCC HTB-92cells) (pre-adipocytes) used for in vitro analysis	-Eplerenone attenuated body weight gain in mice fed with HFD compared to HFD animals (a 47% decrease) -Comparing HFD vs. HFD + Eplerenone there is 48% reduction of fat mass without statistical significance -Adipocyte size markedly increased with HFD vs. normal diet and this effect was lost in the HFD + Eplerenone group - Lipid accumulation in the hepatic lobules in HFD mice markedly increased by HFD and significantly reduced in HFD + Eplerenone. In vitro, Aldosterone induced a shift to the proliferative phase (S + G2M) and decreased the percentage of cells that were in G1 of pre-adipocytes and Eplerenone reversed this effect by modulating the expression of the key commitment markers C/EBPbeta, PPAR-γand HSD11B1	- No significant modification of food intake observed by eplerenone -Plasma levels of aldosterone were significantly increased in HFD vs. normal diet
Marzolla et al. [33]	- C57BL/6 J male mice were fed a normal diet or a HFD (with 60% fat) containing or not Finerenone for 3 months - Inguinal, gonadal, and interscapular tissues were used for histological and immunochemical analysis - For in vivo experiments brown adipocyte cultures (T37i cells) used	-Interscapular BAT histological examination showed that Finerenone treatment induced a significant reduction of lipid droplets size and a significant increase of BAT density compared to HFD group,	-Significant increase in the expression of thermogenesis-related genes UCP-1 PGC1-α,and Adrb3 and significant reduction in the expression of white fat specific markers leptin and adcy5 in HFD + Finerenone group compared to HFD group -In T37i cells, Finerenone increased UCP-1 expression significantly -Finerenone significant increased AMPK activation by inducing phosphorylation at threonine-172 (Thr172), at day 7 of T37i
Maeda et al. [23]	-ApoE-/- mice (C57BL/6 J background) fed with normal chow or Western-type diet randomly divided into Esaxerenone (3 mg/kg/day) or vehicle (carboxymethyl cellulose) groups and treated by oral gavage once daily for 8 or 20 weeks -Cell culture experiments performed in HUVECs -Histologic examination of aorta performed	-In Esaxerenone treated mice, aortic arch showed a significant reduction of lipid deposition compared with vehicle controls	-Esaxerenone decreased macrophages accumulations, expressions of ICAM-1 and VCAM-1 in aortic arch vs placebo In aortic rings, Esaxerenone administration increased eNOS^Ser117^ and AKT phosphorylation -In HUVECs, Esaxerenone attenuated the increase in eNOS^Thr495^ phosphorylation induced by aldosterone in a dose-dependent manner

HFD: high-fat diet, UCP1: uncoupling protein 1, MRAs: Mineralocorticoid receptor antagonists, PGC1-α, peroxisome proliferator-activated receptor g coactivator 1-α, Adrb3: beta-3 adrenergic receptor, A CIDEA: DNA fragmentation factor, a subunit-like effector, FATP: fatty acid transport proteins, Fabp1: Fatty acid-binding protein-1, IL-6: Interleukin 6, TNF-α: tumor necrosis factor-α, MMP: matrix metalloproteinase, FFA: Free Fatty acids, BAT: brown adipose tissue, MCP-1: Monocyte Chemoattractant Protein-1, PPARγ: Peroxisome proliferator-activated receptor gamma, TBARS: Thiobarbituric acid reactive substances, GPAT1: glycerol-3-phosphate acyltransferase-1, DGAT1: DAG acyltransferase-1, TAG: Triacylglycerol, SREBP-1c: sterol response element binding protein-1c, NASH: Nonalcoholic steatohepatitis, Col1α1: collagen-α1, Col3A1:Collagen alpha-1(III), WAT: white adipose tissue, NF-κB nuclear factor kappa-light-chain-enhancer of activated B cells, CEBP-β: CCAAT/enhancer-binding protein beta, HSD11B1: hydroxysteroid 11-beta dehydrogenase B1, BAT: Brown adipose tissue, Adcy5: Adenylate cyclase 5, AMPK:AMP-activated protein kinase, HUVECs: Human umbilical vein endothelial cells, ICAM-1: Intercellular Adhesion Molecule 1, VCAM-1:Vascular Cell Adhesion Molecule-1, eNOS: Endothelial nitric oxide synthase.

**Table 2:** Summary of clinical studies specifically investigating the impact of mineralocorticoid receptor antagonists on fat mass and related mechanisms

References	Methods	Effect on fat mass	Related findings in adipose tissue
Polyzos et al. [27]	-Single-center, 52-week, open-label, randomized controlled trial (RCT) -Participants randomized to receive either oral vitamin E (400 IU/d in 2 equal doses: group 1) or spironolactone (25 mg once daily) plus vitamin E (400 IU/d in 2 equal doses; group 2) for 52 weeks -Thirty-one (23 women) participants with NAFLD (15 with simple steatosis and 16 with NASH) were randomly assigned to group 1 (n:7; 11 women) or group 2 (n:14; 12 women)	-NAFLD liver fat score significantly decreased only within group 2 -Steatosis, NAFLD liver fat score, decreased only within the combination group,	-Both insulin and insulin resistance decreased at 8 weeks with the combined regimen, but not with vitamin E monotherapy
Thuzar et al. [30]	-Randomized double-blind, cross-over designed trial, -two men and eight women underwent 2 weeks of spironolactone (100 mg/d) treatment and placebo, with an intervening 2-week wash-out period -BAT function was assessed in response to cooling and to a mixed meal -Metabolic activity was measured by fluoro-deoxyglucose (FDG) uptake (maximal standardized uptake value, SUVmax) using PET-CT -Thermogenic activity was measured by skin temperatures overlying supraclavicular BAT depots using infrared thermography -Postprandial metabolism was measured by energy production rate and lipid synthesis using indirect calorimetry	-Spironolactone treatment increased BAT activity in 8 of 10 subjects Compared to placebo, mean BAT metabolic activity were significantly higher during spironolactone treatment	-Supraclavicular temperatures increased during both treatments in response to a mixed meal -The postprandial increase in temperature was over three times higher during Spironolactone treatment -Spironolactone increased the proportion of BAT-positive scans from 60 to 90% in 10 participants, and increased FDG uptake by over 50% and BAT volume by over 200%
Johansen et al. [28]	-26-week, double-blind, randomized, placebo controlled trial including patients with Type 2 DM -Patients were randomized 1:1 to either Eplerenone (N:70) with a target dose of 100 or 200 mg/day according to eGFR -65 patients in Eplerenone group and 64 patients in placebo group completed the treatment -The primary outcome measure was change in liver fat by proton magnetic resonance spectroscopy at week 26 from baseline;	-No changes observed in liver fat in the Eplerenone group or the placebo group) -No difference in change from baseline between the groups in abdominal fat distribution, measured as visceral fat volume and subcutaneous fat volume using MRI	-No differences were found in plasma adiponectin, FFA or glycerol levels -No change from baseline in hsCRP was observed with Eplerenone treatment
Fu et al. [29]	-Epicardial or subcutaneous fat obtained from 71 patients undergoing heart surgery -DEXA used for body fat mass determination	-The MRA intake was the main factor associated with lower levels in epicardial fat	-MRA upregulated anti-inflammatory marker intelectin 1 and reduced the proliferation of epicardial fibroblasts -MRA intake associated with a lower level of fatty acid transporters, FABP4 and CD36, in epicardial fat
Ishikawa et al. [31]	-Randomized prospective study comparing the effects of Spironolactone, and Esaxerenone, on sex hormone levels and body composition in patients with primary aldosteronism -Randomization in a 1:1 ratio to receive either spironolactone (n:33) or Esaxerenone (n:32) -Spironolactone (25, 50, or 100 mg) or Esaxerenone (1.25, 2.5, or 5 mg) was administered to achieve optimal BP	-Body fat and muscle mass tended to increase and decrease, respectively, in both groups, and these changes were similar between the two groups	-Serum free testosterone was significantly higher in the Spironolactone vs. Esaxerenone group in both males and females -Estradiol, progesterone, luteinizing hormone, and follicle stimulating hormone same between groups Serum potassium higher in the Spironolactone vs. Esaxerenone group

NAFLD: Nonalcoholic Fatty Liver Disease, NASH: Nonalcoholic steatohepatitis, BAT: Brown adipose tissue, eGFR: estimated glomerular filtration rate, hsCRP: High-sensitivity C-reactive protein, FABP4: fatty-acid-binding protein 4.

The clinical studies specifically addressing the impact of MRAs of fat mass is scarce with more heterogeneous results. Some studies showed that liver fat content decreased with MRAs [27], others could not showed this effect [28]. On the other hand, MRA use was associated with lower epicardial fat [29]. Increased browning of adipose tissue was demonstrated by MRAs [30] Interestingly, other studies showed that there is tendency of increased adiposity and decreased muscle mass with use of MRAs [31].

## Discussion

Obesity is a non-communicable disease results from energy intake in excess to energy expenditure and excess energy causing fat deposition in adipose and-non adipose tissue. Obesity is associated with inflammation, oxidative stress, mitochondrial dysfunction and insulin resistance which may affect distant organs apart from adipose tissue**.** Each of these pathologic events, are interrelated causing additive and synergistic effect [34]. Obesity management starts with behavioral modifications (e.g. nutritional interventions, increased physical activity, psychological support and stress reduction), with the possible addition of obesity management medications, and metabolic or bariatric (surgical and endoscopic) procedures [35]. Fortunately, the medications to treat obesity has been steadily increasing in recent years [36–40].

Steroidal MRAs include spironolactone and eplerenone and non-steroidal MRAs include finerenone and esaxerenone, apararenone, ocedurenone [41] and these medications showed renal and cardiovascular benefits [42]. Various randomized studies have already shown that non-steroidal MRAs have cardiovascular and kidney benefits [43–48]. These beneficial effects can be explained partly by their potential favorable impact on lipid deposition and lipid metabolism (Tables 1 and 2). The rationale of these findings are explained below.

### How did MRAs impact fat mass and lipid metabolism

**Fig. 1:**
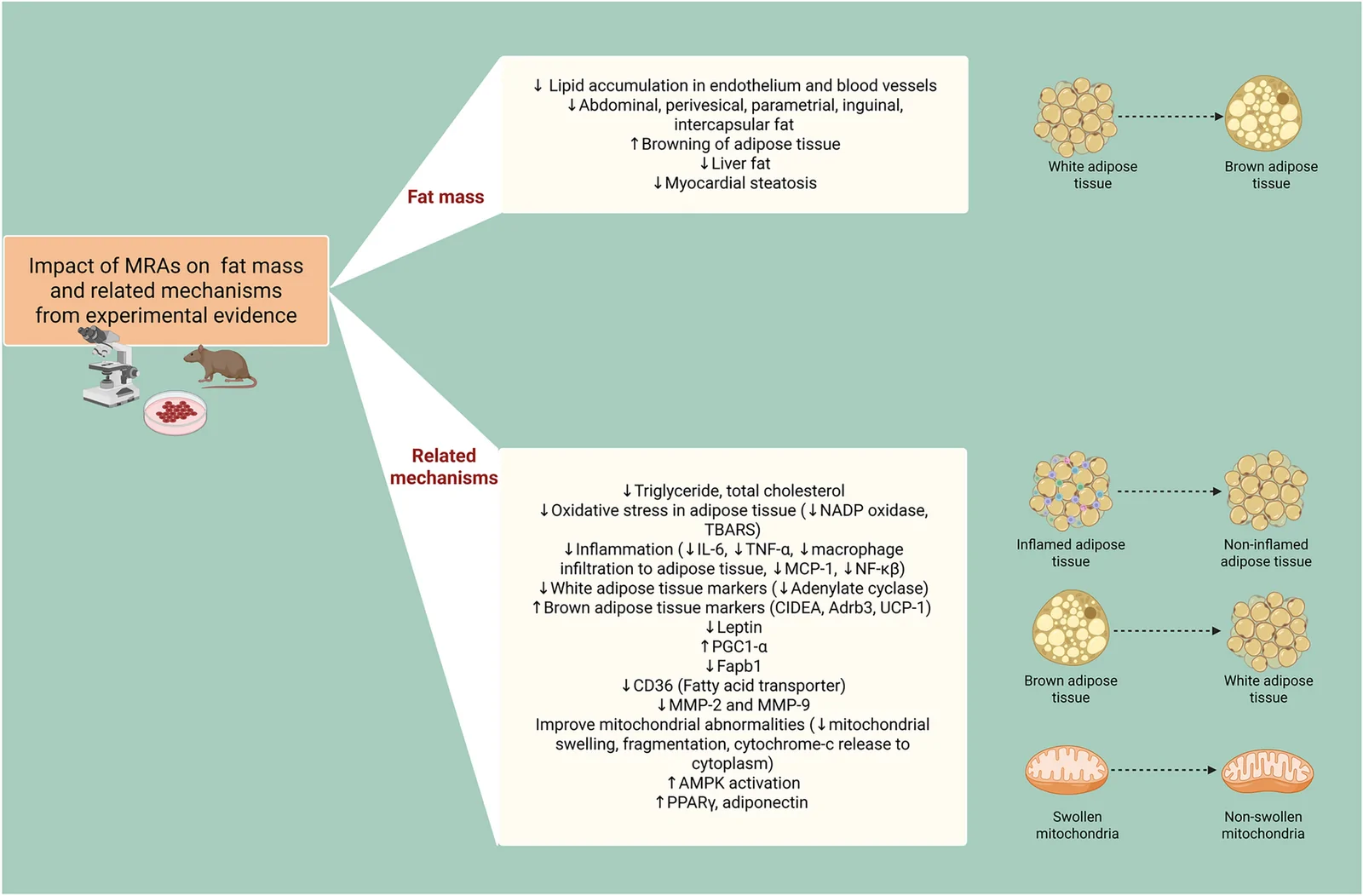
Impact of mineralocorticoid receptor antagonists on fat mass and related mechanisms from experimental evidence

The recognition of adipose tissue as a metabolically active endocrine organ has refined the current understanding of obesity-related complications. Fat accumulation is an active process and contributes to organ-specific dysfunction through pro-inflammatory cytokine release, dysregulated lipid metabolism, and impaired insulin signaling. In adipose tissue, there is active production of aldosterone [15, 49, 50]. Additionally, mineralocorticoid receptor expression is increased in adipose tissue of various preclinical models of metabolic disease [5, 51] suggesting that this increase is detrimental. Indeed**,** increased expression of MR in adipose tissue contributes to metabolic syndrome, visceral obesity and weight gain, glucose intolerance, and insulin resistance, as well as dyslipidemia [52]. On the other hand, increased mineralocorticoid action has ability to increase fat mass in various experimental models and there is bi-directional and amplification loop between fat mass and mineralocorticoid action. To be said, adipocytes secrete mineralocorticoids in conditions of metabolic dysfunction. The increased mineralocorticoid action increases fat mass and further augmenting mineralocorticoid production by adipose tissue. It is very important to break this vicious cycle since the high aldosterone production is related with insulin resistance, inflammation, oxidative stress during obesity [53]. In this context, it is hypothetically beneficial to inhibit adipocyte derived aldosterone synthesis. Indeed, studies have shown that MRAs decrease fat mass in various experimental models and studies showed that treatment with MRAs decreased fat content in endothelium [18] in inguinal and intersacpular area [19], in liver [20–22], in aortic arch [23], in muscle [24], in myocardium [25] and in pancreas [54] which are associated with decreased inflammation, oxidative stress, decreased insulin resistance, decreased apoptosis and improving mitochondrial dysfunction (Fig. 1). The important question remains here: How did MRAs decrease fat mass in adipose tissue? There are some mechanisms to explain this effect.

The first mechanism is to decrease free fatty acid (FFA) uptake into adipocyte. The entry of FFA inside the adipocyte is a regulated process involving various receptors. Plasma membrane fatty acid binding protein (FABPpm), fatty acid translocase (FAT/CD36), fatty acyl CoA synthetases (FATP and ACSL) are all involved in entry of FFA [55]. Habibi et al. showed that western diet increased expression of hepatic fatty acid transporter protein CD36, fatty acid transport proteins (FATP), as well as fatty acid-binding protein-1 (Fabp1) which all prevented by spironolactone [20] Fu et al. demonstrated that epicardial fat biopsies from patients who were taking MRAs expressed lower levels of CD36 and FABP4 [29]. Thus, these evidence suggest that MRAs decrease adipocyte uptake of FFA for synthesis of triglycerides into adipocytes and decrease adipocyte fat production.

The second mechanism is on Leptin. The relationship between leptin and adiposity is complex and paradoxical. At one hand, leptin increases lipolysis and decreases fat synthesis independently of its effects on food intake [56]. However, in vitro studies showed that, leptin had proadipogenic effects at physiological concentrations (10 nM) increasing proliferation and differentiation of primary cultured subcutaneous pre-adipocytes. These changes were associated with increased expression of differentiation markers (LPL and glycerol-3-phosphate dehydrogenase) and two key adipogenic transcriptional factors C-FOS and PPARγ2, together with increased fat storage in these cells [57]. Leptin treatment at concentrations of 8 or 80 ng/mL had similar effects on the murine lineage of pre-adipocytes 3T3-L1 and in primary mouse adipose tissue-derived stromal cells which increased the expression of the adipogenesis and lipogenesis-related proteins PPARγ, SREBP1C, Perilipin 1 (PLIN1), and caveolin 1 (CAV-1) [58]. Other studies shown that a relatively low concentration of leptin (50 ng/ml) stimulates proliferation of preadipocytes from rats in primary culture, whereas leptin at higher concentrations (250 and 500 ng/ml) inhibits proliferation of both preadipocytes and stromal vascular cells [59]. Even more conflicting, high dose of leptin of 1000 ng/ml has been shown to stimulate the proliferation of porcine preadipocytes [60]. It was suggested that the leptin increased lipolysis and decreasing fat synthesis may be irrelevant during obesity due to leptin resistant state as an explanation of this paradox [59, 61]. Previous studies showed that MRAs have decreased leptin levels in adipose tissue [19, 26]. Thus by decreasing leptin levels MRAs may decrease fat mass during obesity.

The third mechanism is on cell proliferation. In vitro, Aldosterone induced a shift to the proliferative phase (S + G2M) and decreased the percentage of cells that were in G1 of preadipocytes and Eplerenone reversed this effect by modulating the expression of the key commitment markers CCAAT/enhancer-binding protein beta (CEBP-β), Peroxisome proliferator-activated receptor gamma (PPAR-γ) and hydroxysteroid 11-beta dehydrogenase B1 (HSD11B1) [16].

### What are the metabolic consequences of MRAs in adipose tissue?

The above discussion suggested that MRAs decrease fat mass by variety of mechanisms. However, apart from fat mass reduction; MRAs also showed various metabolic alterations in adipose tissue. These are summarized in Table 1 and include decrease in inflammation, oxidative stress, and apoptosis in adipose tissue. Furthermore, MRAs improve mitochondrial function and AMP-activated protein kinase (AMPK) signaling which all are relevant during obesity. However, there is another very important effect of the MRAs: browning of the adipose tissue.

As well known, adipose tissue is not homogeneous but composed of white, brown and beige or brite (brown in white) which have distinct functions [6]. WAT is found throughout the body and is classified into two groups: visceral WAT (vWAT) depot and subcutaneous WAT (sWAT) depot [62]. BAT is mainly composed of brown adipocytes that contain large amounts of multilocular/small lipid droplets of various sizes. Brown adipocytes are small and polygonal, unlike white adipocytes. The activity of BAT is decreased in various pathological states such as obesity, diabetes, and aging. Beige adipose tissue is a hybrid form of adipose tissue that shares characteristics with WAT and BAT [63]. During obesity, hyperplasia, hypertrophy, secretion of vasoconstrictors, and immune cell infiltration are observed in WAT [64–66].

BAT preserves body temperature by a process known as non-shivering thermogenesis, dissipating energy in the form of heat. Thermogenesis is mediated through brown adipocyte-specific uncoupling protein 1 (UCP1) [67, 68]. In recent years, there is a growing interest of BAT for management of obesity due to anti-obesity action of BAT [69, 70]. Increased activity of BAT can lead to increased improved glucose and lipid metabolism, energy expenditure, and better control of body weight and metabolic parameters [71]. In this context, various studies showed that MRAs increase BAT activity with increased UCP1 and thermogenic activity [19, 20, 26, 30, 33] favoring action on dissipating energy and obesity.

### Controversies and unknowns

Based on previous discussion, it can be speculated that MRAs are effective in reducing fat mass with beneficial metabolic effects. However, one must be very cautious before reaching firm conclusions. Firstly, the results of the preclinical studies are not translated hundred percent into clinical studies. Also the number of clinical studies specifically investigating the impact of MRAs on fat mass is scarce, and the findings are heterogeneous. The studies are different with respect to included patient’s phenotypes, dosage and duration of treatments, outcome measurements (Table 2). While some studies showed promising results of MRAs [27, 29, 30] others did not demonstrate any benefit [28, 31]. The reasons for the negative findings are not clear however patients were on metformin, GLP-RAs and SGLT-2 which may affect the fat mass already. Furthermore, biopsy was not performed in both studies [28, 31]. The second important issue is the antagonistic actions of steroidal MRAs on androgen receptors and agonistic actions on progesterone receptors [31] which may cause loss of lean body mass. Wada et al. showed that Eplerenone did not affect lean body mass [17]. Ishikawa et al. did not find any effect of spironolactone on muscle mass [31]. However, literature is very scarce in this regard and more studies are needed. Third, the impact of MRAs induced fat mass and metabolism changes on hard outcomes are not known. Does reducing fat mass play a role in cardiovascular protection of MRAs? To the best of our knowledge, no study investigated this issue. Fourth, whether the amelioration of inflammation by MRAs impact fat deposition is not known fully. A recent study showed that eplerenone reduced TNF-α expression in the pancreatic acini and blood vessels and decreased pancreatic pancreatic steatosis [54]. In rats with type 2 diabetes, Finerenone decreased TNF-α and IL-17 along with decreased cardiac steatosis [72]. In C57bl6 mice, Eplerenone reduced hepatic steatosis and inflammation [22]. On the other hand, in db/db mice, although finerenone (10 mg/kg-body p.o.) decreased expression of MCP-1 and macrophage infiltration in perirenal fat tissue; it did not affect adipocyte size [73]. Thus, it needs to be determined that whether suppression of inflammation by MRAs plays a role in attenuation of adiposity. Lastly, one study showed that nonsteroidal MRA antagonist esaxerenone increased body fat percentage in females but not in males. In addition, the muscle mass percentage decreased in females in both spironolactone and esaxerenone groups but not in males. In sub-group analysis of postmenopausal females, spironolactone and esaxerenone did not affect Body fat percentage and muscle mass percentage [31]. The reasons for this findings are not clear but may imply the hormonal differences between males, females and post-menopausal females.

## Future directions

As above discussion suggest, there is accumulating evidence showing that MRAs may have favorable actions on lipid metabolism and lipid accumulation. However, there is knowledge gaps and new research is warranted. Firstly, future randomized studies should consider whether impact of MRAs on lipid metabolism and lipid accumulation on hard outcomes such as cardiovascular and kidney diseases and mortality. Second, weather related mechanisms such as decrease of inflammation play a role in fat metabolism needs to be addressed. Third, the differential impact of MRAs on males and females or postmenopausal females as shown in one study [31] should be investigated more deeply. Fourth, specific studies are needed to highlight the impact of MRAs on specific fat depots (visceral vs. subcutaneous) or different patient populations (e.g., obesity, sarcopenic obesity).

## Conclusions

Both steroidal and non-steroidal MRAs are used for cardiovascular and renal protection. In addition, recent research suggests that MRAs also decrease fat mass and improve lipid metabolism. Currently, the related mechanisms are not clearly known. Based on the encouraging preliminary findings, more preclinical and clinical studies are needed to investigate these issues.

## Data Availability

This is a narrative review; no new scientific data was generated, and all data are within the paper.
